# 3D Titania Nanofiber-Like Webs Induced by Plasma Ionization: A New Direction for Bioreactivity and Osteoinductivity Enhancement of Biomaterials

**DOI:** 10.1038/s41598-019-54533-z

**Published:** 2019-11-29

**Authors:** Mohammad-Hossein Beigi, Naghmeh Safaie, Mohammad-Hossein Nasr-Esfahani, Amirkianoosh Kiani

**Affiliations:** 1 Silicon Hall: Micro/Nano Manufacturing Facility, Faculty of Engineering and Applied Science, Ontario Tech University, Ontario, Canada; 2 Department of Cellular Biotechnology Cell Science Research Center, Royan Institute for Biotechnology, ACECR, Isfahan, Iran

**Keywords:** Cell biology, Engineering, Materials science, Optics and photonics

## Abstract

In this study, we describe the formation method of web-like three-dimensional (3-D) titania nanofibrous structures coated on transparent substrate via a high intensity laser induced reverse transfer (HILIRT) process. First, we demonstrate the mechanism of ablation and deposition of Ti on the glass substrates using multiple picosecond laser pulses at ambient air in an explicit analytical form and compare the theoretical results with the experimental results of generated nanofibers. We then examine the performance of the developed glass samples coated by titania nanofibrous structures at varied laser pulse durations by electron microscopy and characterization methods. We follow this by exploring the response of human bone-derived mesenchymal stem cells (BMSCs) with the specimens, using a wide range of *in-vitro* analyses including MTS assay (colorimetric method for assessing cell metabolic activity), immunocytochemistry, mineralization, ion release examination, gene expression analysis, and protein adsorption and absorption analysis. Our results from the quantitative and qualitative analyses show a significant biocompatibility improvement in the laser treated samples compared to untreated substrates. By decreasing the pulse duration, more titania nanofibers with denser structures can be generated during the HILIRT technique. The findings also suggest that the density of nanostructures and concentration of coated nanofibers play critical roles in the bioreactivity properties of the treated samples, which results in early osteogenic differentiation of BMSCs.

## Introduction

Although, bone autograft and allograft are considered the gold standard for bone regeneration, the associated risk of donor site morbidity and limited supplements remain disadvantages of these methods. Tissue engineering approaches, as a promising alternative, aim to facilitate bone regeneration, even in large craniofacial skeletal defects^1–4^.

Among different tissue engineering methodologies, bone tissue engineering with the support of stem cells is of great interest considering their ability to differentiate into a variety of cells and their self-renewal ability^5^. Human mesenchymal stem cell (hMSCs) differentiation is an ethical way of tissue remodeling. Simply, cell and Extracellular Matrix (ECM) interaction is mutual: cells affect and are affected by ECM^6,7^. Indeed, the physical properties of surfaces that ECM forms, such as structure, topography, and physical and chemical properties, influence MSC livability and self-restoration, differentiation, and proliferation^7^. MSCs, which dwell in the bone marrow and other tissues, are able to regenerate damaged tissues when employed in specific scaffolds and implanted into defect sites^8^. Cultured-cell to ECM and their adjacent cell interaction plays a crucial role in cell viability and adhesion, proliferation, migration, and differentiation, as well as bone remodeling and osseointegration. These two factors (materials and cells) will be completed by a preferable fabrication method as the third side of the bone tissue engineering, which can provide a desirable platform for these to work together. Several methods are currently used to produce bone tissue engineering surfaces including sol-gel (20), hydrothermal 106^9^, electrospinning^10^, solvent casting^11^, 3D printing^12^, etc.; however, selecting the most suitable method remains the main challenge. Artificial biomaterials used in tissue engineering, need to react with physiological fluids and form tenacious bonds to hard and soft surrounding tissues through cellular activity. Thus, they must be compatible with biologic organs and have good connections with them. They should also be able to conduct complex mechanisms of ion exchange with body fluids and motivate multi-potent mesenchymal stem cell differentiation and maturation^3,4^. Titanium and its alloys are among the most useful biomedical metals widely used in orthopedic implants^13^. It has been shown that titanium nanoparticles (NPs) promote the osteogenesis of dental pulp stem cells (DPSCs) and adipose-derived stem cells (ADSCs)^14^. In addition, the osteogenic differentiation of BMSCs on TiO_2_ surface was more effective compared to the cover glass^15^. TiO_2_ hydrothermal-treated surface with a flake-like topography and high porosity can ultimately enhanced osteogenesis^9^.

Laser surface modification is a novel film deposition technique that can be used to modify the surface’s absorption, adsorption, chemistry, roughness, biocompatibility, and bioreactivity of biomaterial surfaces, which can reduce the chance of failure after implantation in the body^16–18^. According to our previous publication^17^, HILIRT can be employed to deposit various materials on glass substrates, which can potentially be used for the production of lab-on-a-chip components and other forms of biomaterials^17,18^. HILIRT enables us to deposit nano fibrous titania (NFTi) thin-film on a substrate. In this chemical-free process, high laser energy pulses in the range of 30 ns to 150 ps are transmitted through the transparent substrate and focused on target material placed 300 µm from the substrate. During the pulse ionization process, titanium is ablated and deposited on the back of the substrate in the form of nanofibrous structures^17^. By altering the laser parameters, mechanical, biological, and corrosion behaviors of substrate, such as roughness, ion release, protein adsorption/absorption, and cell viability, the differentiation process can be modified^17–19^.

**Figure 1:**
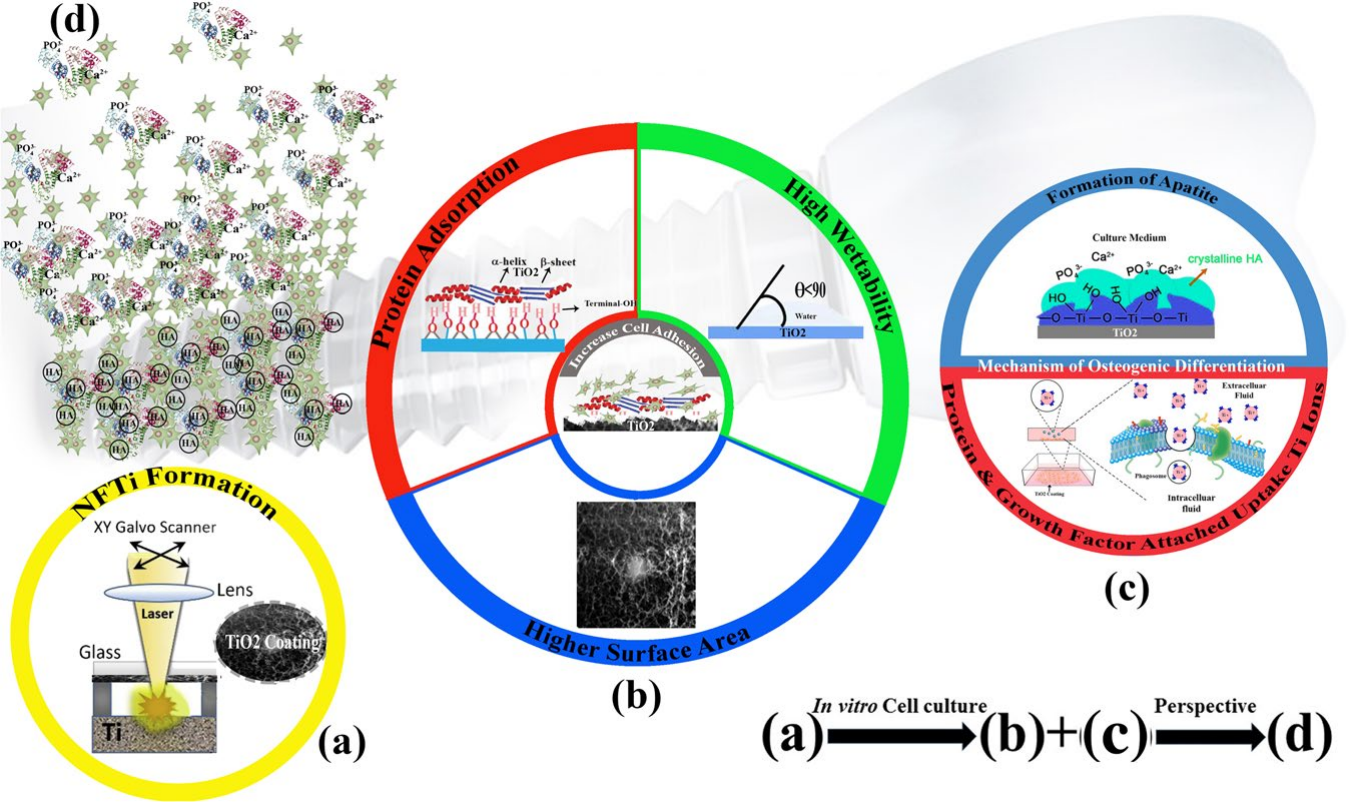
Schematic mechanisms of cell proliferation and osteoinductivity of nanofibrous titanium coating by surface modification through high intensity laser induced reverse transfer (HILIRT): A novel deposition method. (**a**) NFTi layer deposited on glass by the proposed HILIRT technique at laser beam scanning speeds. (**b**) The biocompatibility of titanium as an implant material is attributed to surface oxide spontaneously forming in air and/or physiological fluids, and it is believed that cellular behaviors, e.g., adhesion, spreading and proliferation are greatly affected by: 1. Surface area 2. wettability 3. surface hydroxyl groups (The surface hydroxyl groups of terminal OH^-^ regulate the initial protein adsorption behaviors). (**c)** Surface hydroxyl groups and bioactive Ti nanoparticles promote osteoblast differentiation through 1. The Ti-OH groups formed on the surface of titanate after soaking in osteogenic culture medium are negatively charged, and hence combine selectively with the positively charged Ca^2+^ ions in the fluid to eventually form calcium phosphate. 2. Biocomplexes (ions, protein and growth factor) are internalized by caveolae mediated endocytosis. (**d)** Perspective: Bone formation and remodeling around implanted materials.

In this research, we investigated the effect of laser pulse duration in the HILIRT method on materials and biological behaviors of synthetic biomaterials substrate via materials characterizations and *in-vitro* analysis. The NFTi thin film was soaked in simulated body fluid (SBF) to form the Hydroxyapatite (HA)-like layer structure on the substrate. Materials and structural properties and identification of the generated NFTi and the formed HA-like layer were analyzed using water contact angle (CA), scanning electron microscopy (SEM), Energy Dispersive X-Ray (EDS) analyzer, micro-Raman and X-ray (XRD) spectroscopies. In order to study the effects of laser pulse duration on the proliferation and osteogenic differentiation of human bone marrow-derived mesenchymal stem cells in contact with the specimens, *in vitro* analyses including MTS assay, immunocytochemistry, mineralization, ion release examination, gene expression analysis, and protein adsorption and absorption were conducted (schematic Fig. 1).

## Materials and Methods

### Materials

Focused picosecond and nanosecond laser pulses were transmitted through glass and focused on the surface of the Grade 4 Ti substrate. The pulse ionization process for deposition of ablated Ti to the glass substrate was done by an Ytterbium pulsed fiber laser system (IPG Laser Model: YLPP-1-150V30) at a wavelength of 1064 nm. Sample preparations were done with a constant power of 9 W, a pitch of 0.025 mm, and a scanning speed of 100 mm/s at a frequency of 600 kHz and at 150 ps, 5 ns, and 30 ns pulse duration under ambient conditions. Samples were soaked in SBF for four days at 37 °C as described^17^. The SBF used in this research is a solution with similar ion concentration to human blood plasma, which was prepared according to Kokubo, T. and Takadama, H., 2006 protocol^20^.

### Materials characterization

#### Imaging and spectroscopy

In order to analyze the morphology and material properties of the deposited Ti thin-film structures and the HA-like mineral layer deposited on generated thin film after soaking in SBF, a Scanning Electron Microscope (SEM) and an EDAX Genesis 4000 Energy Dispersive X-Ray (EDS) analyzer were used. A Rigaku Ultima IV X-ray diffractometer (XRD) using Cu Kα radiation (λ = 0.15418 nm, 40 kV and 44 mA) and a Renishaw inVia Raman spectrometer (514 nm argon ion laser with a 25 mW power; repeated acquisitions: 40 scans with an acquisition time of 10 s at a 50 × magnification) were utilized for the structural identification of deposited titania coatings and HA-like layers formed on them.

#### Contact angle

The contact angle (CA), where a liquid-vapor interface meets a solid surface, describes the ability of a liquid to be in contact with a solid surface (wetting) through Young equations. In this experiment the CA tests for cell attachment potential of specimens were performed utilizing 5 μl of distilled water droplets dropped from distance of 1 cm and recorded 5 seconds after contact with the fabric surface using a self-developed goniometer apparatus with a high-resolution camera^21^. The average value of 3 replicates measured on both sides of the drops was reported as the CA of each sample.

### Cell culture and biology evaluation

Cell culture studies with human bone-derived mesenchymal stem cells (BMSCs) were performed for investigation on cell-coating interaction and differentiation. Accordingly, bone fragments obtained during orthognathic surgeries were placed over mesh covered with 4-(2-hydroxyethyl)−1-piperazineethanesulfonic acid (HEPS) medium and centrifuged at 2500 rpm to force out bone marrow cells. The cells were seeded on 25 cm^2^ flasks containing alpha-modified Eagle’s medium (αMEM) supplemented with 15% fetal bovine serum (FBS), L-glutamine, non-essential amino acids (NEAA) and antibiotics (penicillin 0.1 g/L; streptomycin 0.1 g/L) at 37 °C in a humidified air atmosphere containing 5% CO_2_. Upon 80–90% confluence, these cells (detached with 0.05% trypsin-EDTA) were considered as passage zero (P0). P0 BMSCs were cultured and the medium was changed to fresh media every 2–3 days, at 70–80% confluency, the cells were detached using 0.25% trypsin-1 mM EDTA-4Na and seeded on NFTi coated glass and control groups at 5,000 cells per well in 48‐well culture dishes. The culture medium was renewed every 3 days. (All experiments were performed using cells at passages 3–6). All chemicals and reagents, unless otherwise stated, were purchased from Sigma Aldrich (St. Louis, MO). Human bone fragments were donated by healthy male donors (two volunteered patients aged 20–30) who had no systemic complications and were undergoing oral and maxillofacial surgery at Isfahan Medical University, Isfahan, Iran. All procedures and manipulations were approved by the Ethical Committee of Isfahan Medical University, Iran (IR.MUI.REC.1395.4.040). All the individuals who donated tissue signed consent forms.

#### MTS assay

A metabolic activity (MTS) assay was done in order to study the metabolic activity on NFTi coated glass as well as cell adhesion/proliferation and cytotoxicity assessments of BMSCs as compared to tissue culture plate (TCP) and uncoated glass after 4 hours and 2, 4, and 6 days of BMSCs seeding. In this colorimetric quantification method, cell metabolic activity is justified with a colored formazan product which is soluble in cell culture media in a humidified atmosphere containing 5% CO_2_ at 37 °C, by MTS tetrazolium compound reduction. The MTS assay was treated by direct contact with NFTi coated glass with 5,000 human bone-marrow-derived mesenchymal stem cells in each of 48 wells. After 4 hours and 2, 4 and 6 days after cell seeding, cells were washed with phosphate buffered saline (PBS) and incubated with a fresh medium of 20% CellTiter Aqueous One Solution Cell Proliferation (MTS reagent by Promega, Madison, Wisconsin, USA) comprising serum-free medium in the dark at 37 °C in 5% CO_2_ for 4 hours. Finally, aliquots were pipetted into a 96-well plate and the absorbance content of each well was calculated at 492 nm with a Fluostar Optima, BMG Lab Technologies spectrophotometric plate reader^21^. Results were normalized as the ratio of medium without cells.

#### Immunocytochemistry

The spreading behavior and cytoskeletal arrangement of BMSCs seeded onto the titania coatings were studied by fluorescence microscopy using an Olympus BX61. F-actin is a family of spherical poly-operational proteins that form actin filaments, which configure parts of a cell cytoskeleton. This protein, which is one of the most abundant proteins in the body, can be stained in live cells to specify and track the structure and function of the cell’s cytoskeleton and can be seen with a fluorescence microscopy. After 7 days of culture, cells were washed with PBS and fixed with 4% formaldehyde in PBS for 15 minutes at room temperature. Then, cells were permeabilized in 1% BSA and 0.4% Triton. After washing the permeabilizing solution, F-actin was stained with by phalloidin (actin filament, red colour, 1:300 Gibco) for 2 hours. Finally, the cell nucleus was stained with DAPI (4′,6-diamidino-2-phenylindole) (10:1000, Gibco) for 3 minutes and washed with PBS.

#### Differentiation process

BMSCs were cultured on NFTi coated glass (S1: 150 ps, S2: 5 ns, and S3: 30 ns) to induce osteogenic differentiation in comparison with a TCP and uncoated glass in osteogenic induction medium (10 nM dexamethasone, 50 mg/ml ascorbic acid 2‐phosphate, and 10 mMβ‐glycerophosphate) for 21 days. Cells were cultured at a density of 5,000 cells per well in 48‐well culture dishes in the aforementioned medium at 37 °C, 5% CO_2_ for 21 days based on previous studies^22^.

#### Mineralization

The ability of precipitation of inorganic materials on the organic substrate, which is called mineralization, was investigated on samples to determine the effects of specimens on BMSCs differentiation. After 0, 7, 14, and 21 days, cells were fixed in 4% formaldehyde in PBS for 20 minutes at room temperature and stained with 0.5% alizarin red solution in water (pH = 6.4) for 2 hours. Alizarin red was dissolved in a 1 M HCl solution for 2 hours with shaking. Finally, the color intensity of each group was measured at 575 nm using potent mineralization alizarin red-positive nodules area and calcium colorimetry-based assays with a Fluostar Optima, BMG Lab Technologies spectrophotometric plate reader.

#### Gene expression analysis

Gene expression analysis is one of the tools of gene expression used after cell seeding. Gene expression level in the bone differentiation process was measured using reverse transcriptase-polymerase chain reaction (RT-PCR) in the culture on days 0, 7, 14 and 21. Total ribonucleic acid (RNA) was isolated with TRIZOL reagent (Invitrogen) and an RNAeasy Mini kit (Qiagen, Hilden, Germany) following the manufacturer’s protocol. Total RNA was pretreated with DNase I (Fermentas, Waltham, MA, United States). cDNA synthesis was carried out with 1 μg of total RNA using random hexamer primer and the RevertAid H Minus First Strand cDNA Synthesis Kit (Fermentas, Waltham, MA, United States). RT- PCR was performed using SyBr master mix (TaKaRa) at a final volume of 20-μl in a Corbett real‐time PCR (polymerase chain reaction) system (Rotor‐gene 6000, Australia). The specific primers (collagen type I, osteopontin and osteocalcin) were designed with Oligo designer software version 5.2. All samples were assayed in triplicate and three independent experiments were performed. The analysis of gene expressions was performed by the ΔΔCT method, and the beta actin housekeeping gene was used to normalize the data. Also, in order to confirm the differentiation of BMSCs, the osteogenic-related mRNA relative expression, including RUNX2, Collagen I, Osteopontin, and osteonectin, was measured by qRT-PCR for different samples. Gene expression analysis is shown in Table 1.

**Table 1:** Sequence of Primers for Gene Expression.

Gene	Sequence	Annealing temperature (°C)
RUNX2	F: 5′ ATGACACTGCCACCTCTGA 3′ R: 5′ ATGAAATGCTTGGGAACTGC 3′	58
Collagen I	F: 5′ TAGTCTGTCCTGCGTCCTCTG 3′ R: 5′ TTTTGCTTCCTCCCACCCCTA 3′	57
Osteonectin	F: 5′ TGCTATCATTTGCTGTGGAG 3′ R: 5′ ACTCCGTCTTCTTGATGAT 3′	56
Osteopontin	F: 5′ GCTCATTGCTCTCATCATTGG C 3′ R: 5′ GGCTAAACCCTGACCCATCTC 3	60
GAPDH	F: 5′ CCACTCCTCCACCTTTGACG 3′ R: 5′ CCACCACCCTGTTGCTGTAG 3′	56

#### Degradation: Ion release concentration and cell culture medium pH

One of the methods of analyzing the degradation rate of specimens in a body is by measuring the concentration of ions after days of soaking in simulated culture body media. Soaking or immersion tests can be carried out statically or dynamically. For this research, the ion release test was conducted for 1, 3, 7, 14, and 21 days via immersion of NFTi coated samples in a cell culture medium in static condition without any laminar flowing force; the ion concentrations in cell culture medium were measured after sample removal using the analytical inductively coupled plasma mass spectrometry method. In addition, the cell culture medium pH was measured with an 827 pHlab Metrohm meter after the samples were removed.

#### Protein adsorption and biocomplex adsorption/absorption

The protein adsorption of osteogenic differentiation medium for all of the surfaces without cell (A′) was measured by a NanoDrop (Thermo Scientific 3300) at 528 nm. At the beginning the light absorbance of an osteogenic differentiation-specific medium (sigma) was measured by Nano drop and the calculated amount was observed as 100%. The medium was incubated in contact with the coated, uncoated, and TCP samples at 37 °C for 6 hours. The medium protein concentration (A) was then measured by light absorbance.

$${\rm{Adsorbed}}\,{\rm{protein}}\,({\rm{A}}^{\prime} )\,( \% )=100-{\rm{A}}( \% )$$

To calculate the amount of proteins which form a biocomplex with soluble ions, the medium in contact with the samples was centrifuged at 14000 rpm for 30 min (ref.). This leads to biocomplex precipitation (B′). The supernatant protein concentration was measured by NanoDrop (B)

$${\rm{Biocomplex}}\,{\rm{protein}}\,({\rm{B}}^{\prime} )\,( \% )={\rm{A}}-{\rm{B}}$$

To estimate the biocomplex absorption by the cells, the BMSCs was first cultured on the NFTi coated, uncoated, and TCP samples with the osteogenic differentiation specific medium and then incubated at 37 °C for 6 hours. Then the medium protein concentration was measured by NanoDrop (C). Based on the following equation, the percentage of biocomplex uptake by the cells (C′) can be measured:

$${\rm{Biocomplex}}\,{\rm{uptake}}\,{\rm{by}}\,{\rm{the}}\,{\rm{cells}}\,({\rm{C}}^{\prime} )=({\rm{100}}-{\rm{A}}^{\prime} )-{\rm{C}}$$

and

$${\rm{Adsorbed}}\,{\rm{biocomplex}}={\rm{B}}^{\prime} -{\rm{C}}^{\prime} (\mathrm{data}\,{\rm{was}}\,{\rm{not}}\,\mathrm{shown})$$

### Ethics declarations

#### Statement

The present study was carried out in accordance with the ethical principles of the Declaration of Helsinki.

## Results

**Figure 2:**
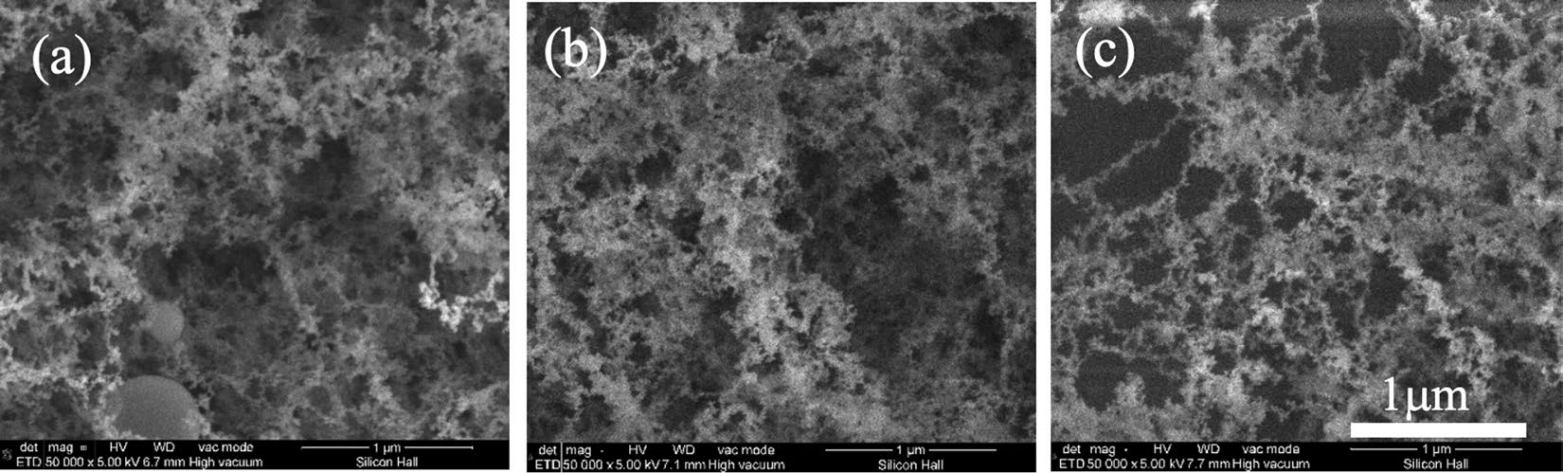
SEM images of NFTi layer with power = 10 W, frequency = 600 KHz (**a**) pulse duration = 150 ps, (**b)** pulse duration = 5 ns, **(c)** pulse duration = 30 ns with 50000X magnification.

**Figure 3:**
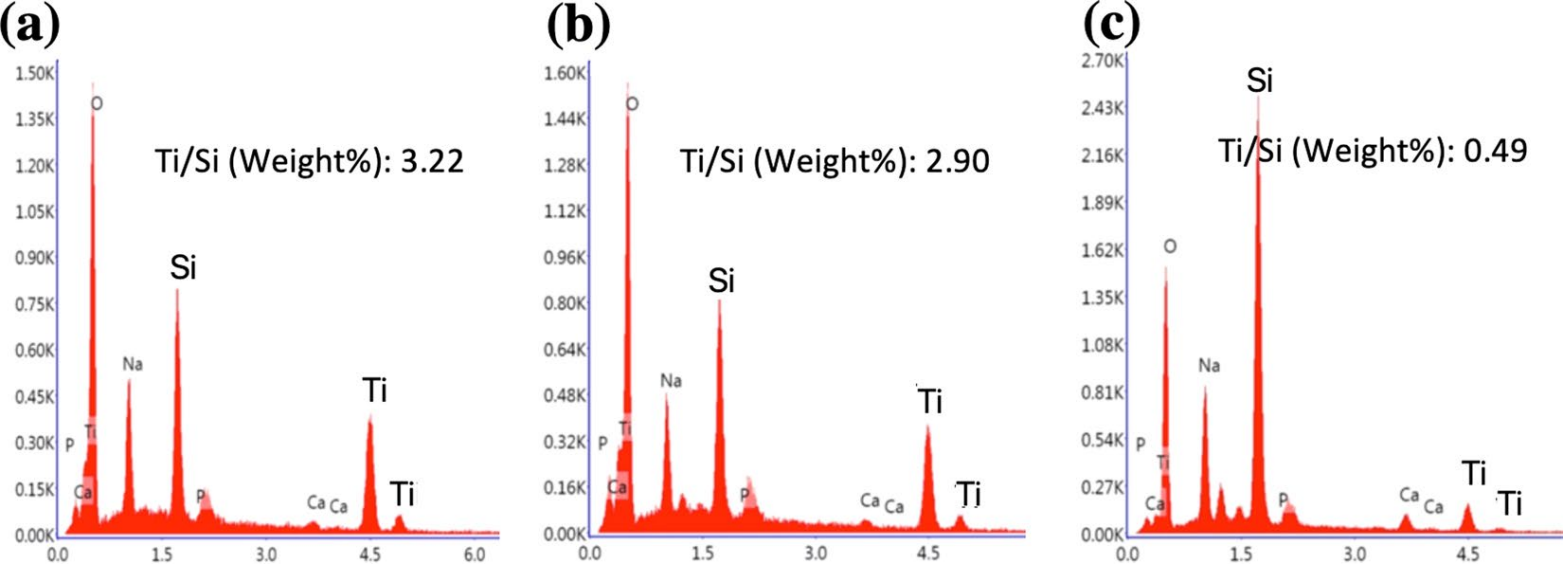
EDX chart for a pulse duration of (**a**) 150 ps, (**b**) 5 ns and (**c**) 30 ns.

**Figure 4:**
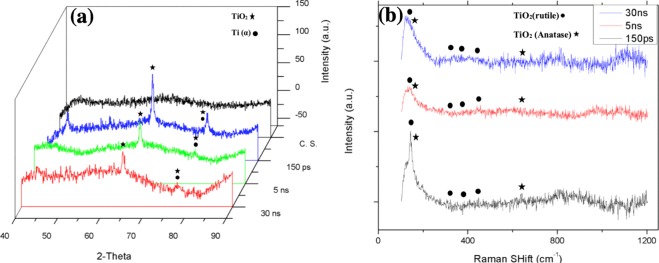
(**a**) XRD pattern, (**b**) Raman spectrum of bare glass and samples coated by titania with different pulse durations (created by Origin Pro 2019B (GF3S4-3089-7907079) https://www.originlab.com/).

Figure 2 shows the deposited NFTi structures at different pulse durations. The low magnification SEM images reported before, show the laser nanofibrous coated surfaces are very smooth (homogeneous)^16–18^. The EDX results in Fig. 3 show the trace of both Ti (deposited layer) and Si (glass substrate) and the Ti/Si ratio at 150 ps, 5 ns, and 30 ns. As shown, by decreasing pulse duration, the titanium weight percentage increased, and the Ti/Si ratio increased by decreasing the pulse duration from 30 ns to 150 ps, which is relevant to the spectroscopy results of both XRD and micro-Raman presented in Fig. 4.

In laser processing of metals, the absorbed laser energy heats up surface atoms to the melting temperature and afterwards to the vaporization temperature. By decreasing pulse duration (τ) the power is transmitted to the target in a shorter time, which causes the heat affected zone (HAZ) to have a higher temperature^23,24^. This results in generation of a plasma plume with a higher temperature containing more particles and ions; thus, a higher amount of NFTi will be deposited on the glass substrate. In shorter pulse durations of the picosecond regime, dense packages of energy in the form of laser pulses are conducted to the substrate electrons on a time scale faster than the transition of energy from those electrons to the lattice of the material, which causes local metal fragmentation rather than the classic heating process. In our research, this process occurred more in samples generated with pulse durations of 150 ps than with samples generated with pulse durations of 5 ns or 30 ns^24^.

The XRD results in Fig. 4 show that the titanium (alpha) and titanium oxide phases reflected severe peaks on the sample created by a laser pulse duration of 150 ps, and weak peaks reflected from samples generated with 5 and 30 ns pulse duration, which is the result of less titania generation on the samples^18^. The micro-Raman results in Fig. 4(b) are in agreement with EDX and XRD results, as generally peaks of both titania phases (rutile and anatase) slightly increase by decreasing pulse duration. Although the electrons of the solid target become prodded by the absorption of laser energy, while a high temperature laser pulse interacts with that target and electrons, energy is quickly conducted to the lattice^25–27^. The temperature gradient can roughly be estimated by Eq. 1 in which r is the radius of the laser spotted area and z is the depth of the laser affected area.

1 $$\varDelta T(r,\,z,\,\tau )=\frac{{{I}}_{max}\gamma \sqrt{\kappa }}{\sqrt{\pi K}}{\int }_{0}^{\tau }{\frac{p(\tau -t)}{\sqrt{t}\frac{8\kappa t}{{W}^{2}}}}^{-[\frac{{z}^{2}}{4\kappa t}+\frac{{r}^{2}}{4\kappa t+0.5{W}^{2}}]}dt$$

In this equation *p(t)* can be assumed with the value of 1 for a theoretical square-shaped pulse at the center point of the HAZ. The calculated temperature profile indicates that a shorter laser pulse duration results in deeper and higher temperatures in the HAZ^25,26^.

**Figure 5:**
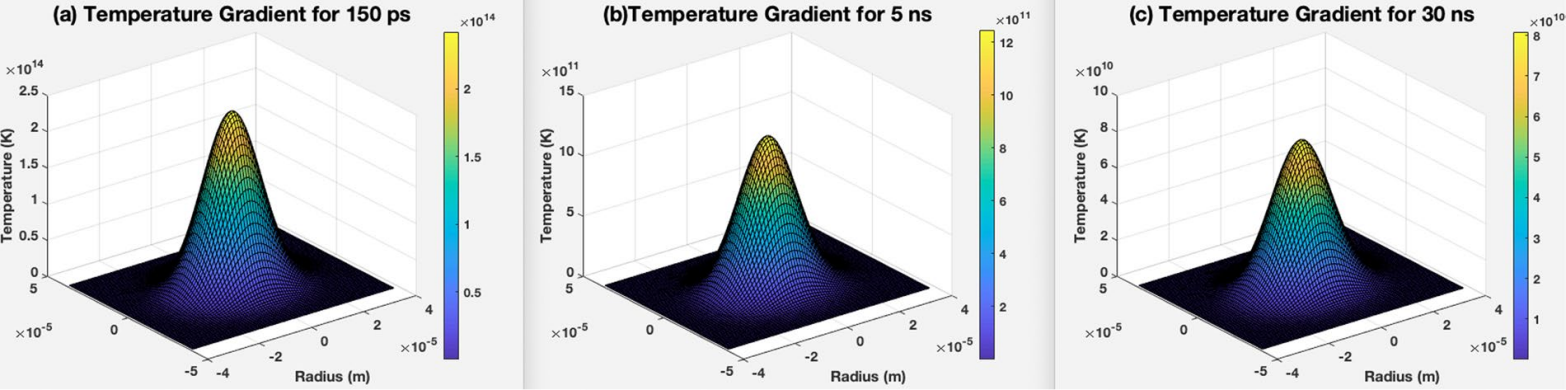
Temperature gradient profile for (**a**) 150 ps pulse duration, (**b**) 5 ns pulse duration, (**c**) 30 ns pulse duration (created in MATLAB R2015b software (9.6.0.1072779) https://www.mathworks.com).

The temperature gradients as a function of *r* and *z* for different pulse durations of 150 ps, 5 ns, and 30 ns are shown in Fig. 5(a–c). The computed results clearly show the temperature of the irradiated zone is significantly higher for a shorter pulse duration of 150 ps in comparison with 5 ns and 30 ns.

The ablation depth as a function of *r*; a radial position from the beam’s center can be calculated as in Eq. 2 ^25^:

2 $$h(r)=\sqrt{-4\beta \kappa \tau \,\mathrm{ln}\{\frac{\beta \kappa \varDelta {T}_{B}}{\gamma {I}_{\max }}\sqrt{\frac{\pi }{\kappa \beta \tau }}(1+\frac{8\beta \kappa \tau }{{W}^{2}}\}-\frac{{r}^{2}}{1+\frac{{W}^{2}}{8\beta \kappa \tau }}}\le h(0)$$

In Eq. 2, Δt_B_ = T_boiling_ − T_room temperature_ (300 K), and *β* constant is about 0.5 which meets valid results for pulse durations up to 300 ns, *k* is titanium diffusivity with the amount of 0.9 × 10^−5^ m^2^/s, and *K* is titanium thermal conductivity, which is equal to 21.9 [W/m.K]. *R* with the value of 0.55 is titanium Fresnel energy reflectivity, and γ is (1 − R) as a fraction of pulse energy absorbed by titanium. *W* is the beam’s field radius (*1/e*) which can be determined through the following equation:

3 $${\rm{D}}=2\times {d}_{f}\times (Msquared)\times laser\,wave\,length/(\pi \times d)$$

In Eq. (3), *d*_*f*_ is the focal distance between the laser lens and the titanium sheet, and *d* is the initial spot size on the titanium substrate, calculated to be around of 20 µm. *I*_*max*_ in Eq. 1, is the maximum intensity, which is calculated with the ratio of measured power to the laser spot area as:

4 $${I}_{\max }=(4{P}_{measured})/(\pi {d}^{2}f\tau ).$$

**Figure 6:**
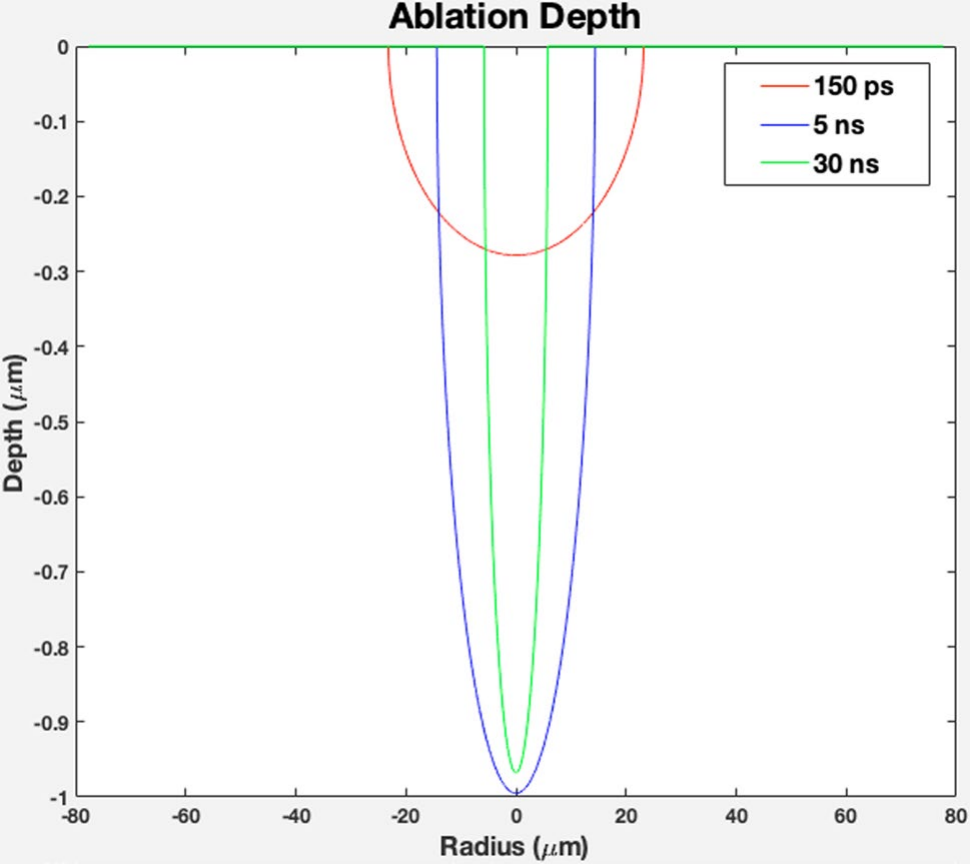
Theoretical ablation depth profile as a function of radius for pulse duration of 150 ps, 5 ns, and 30 ns (created in MATLAB R2015b software (9.6.0.1072779) https://www.mathworks.com).

Here, *f* is the laser pulse frequency, which is 600 kHz, and *τ* is the laser pulse duration (150 ps, 5 ns, and 30 ns). The ablation depth as a function of *r* for 3 different pulse durations is illustrated in Fig. 6. As shown in the graph, the ablated zone for the shortest pulse duration of 150 ps is shallower as we have more heat accumulation and smaller HAZ. The theoretical results presented so far are in agreement with the experimental results, as the shorter pulse duration results at higher ablation temperature lead to generation of a denser plasma plume at a higher temperature; thus, more TiNF structures were formed on the glass substrate in this process.

**Figure 7:**
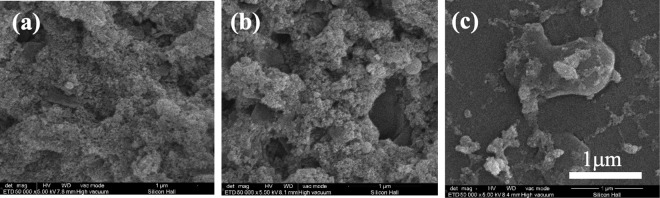
(**a**) XRD pattern, (**b**) Raman spectrum of bare glass and samples coated by titania with different pulse durations after 2 days immersion in SBF, (**c**) XRD pattern, (**d**) Raman spectrum of bare glass and samples coated by titania with different pulse duration after 4 days immersion in SBF Fig. 4. **(a**). XRD pattern, (**b**) Raman spectrum of bare glass and samples coated by titania with different pulse durations (created by Origin Pro 2019B (GF3S4-3089-7907079) https://www.originlab.com/).

The specimens’ bioactivity results in terms of HA-like layer deposition via immersion in SBF after 2 and 4 days are displayed in Fig. 7.

**Figure 8:**
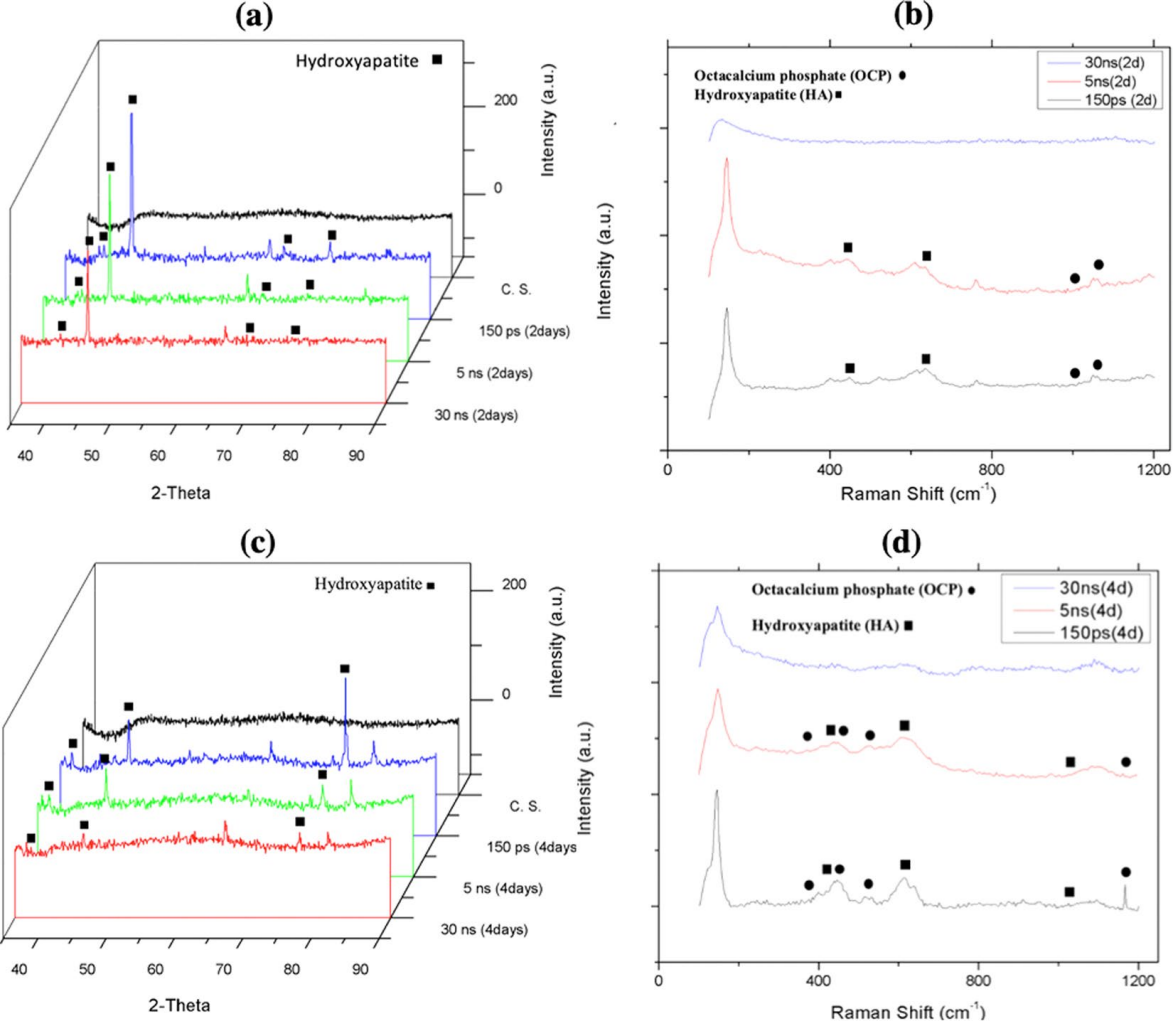
SEM images of NFTi layer with power = 10 W, frequency = 600 KHz (**a**) pulse duration = 150 ps after 4 days immersion in SBF, (**b**) pulse duration = 5 ns after 4 days immersion in SBF, (**c**) pulse duration = 30 ns after 4 days immersion in SBF.

As expected, by increasing laser pulse duration, less HA and other calcium and phosphorous compositions were deposited on the NFTi coated samples^17,28^. This can be the result of less nanofiber generation on NFTi coated glass substrates at a higher laser pulse duration. Decreasing the laser pulse duration means creating more biocompatible Ti nanofibers, which absorb more HA-like content on the samples as shown in Fig. 8. Also, the elevation of HA-like layer peaks in XRD patterns of the corresponding samples in Fig. 7(a,c) and other relevant compositions of calcium and phosphate in the Raman spectrum in Fig. 7(b,d) are in agreement with the XRD results. Decreasing laser pulse duration and eventually creating more NFTi with higher surface-to-volume ratio (SVR) would provide more sites for nucleation and growth of different calcium and phosphate minerals such as hydroxyapatite and octa calcium phosphate (OCP) with a calcium-to-phosphate weight percentage ratio of 1.67 and 1.33 respectively^29^. Consequently, more biocompatibility and less rejection after implanting in the body is possible for the samples prepared at lower pulse duration^17,30^.

### Cell culture, pH

**Figure 9:**
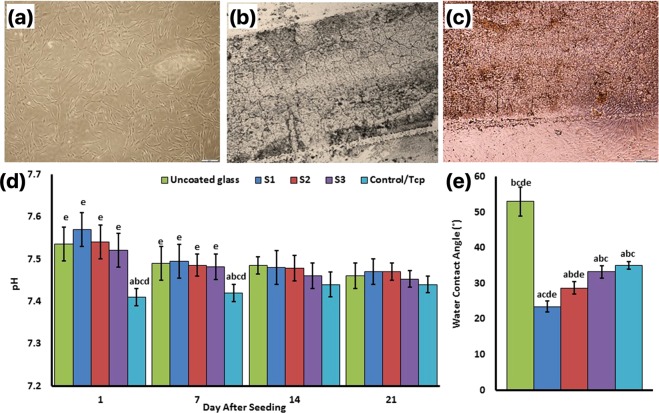
Phase‐contrast microscopy image of Fibroblast-like BMSCs in the medium culture in the third day with 60% confluency (**a**), Titania coats surface on the glass (**b**), and BMSCs interaction with the Ti-coated glass surface with their normal morphology (**c**). The pH of the culture medium before medium refreshment after 1, 7, 14, and 21 days of cell seeding (**d**). Water droplet contact angles of specimens (**e**). (**a**) Statistically significant difference compared with uncoated group at *p* < 0.05; (**b**) statistically significant difference compared with S1 group at *p* < 0.05; (**c**) statistically significant difference compared with S2 group at *P* < 0.05. (**d**) Statistically significant difference compared with S3 group at *p* < 0.05; (**e**) statistically significant difference compared with TCP group at *p* < 0.05.

Figure 9 shows the phase‐contrast microscopy image of Fibroblast-like BMSCs in the medium culture in the third day with 60% confluency (Fig. 9(a)), Titania coats surface on the glass surfaces (Fig. 9(b)), and BMSCs interaction with the NFTi coated glass surface with their normal morphology (Fig. 9(c,d) indicates the pH of the culture medium before medium refreshment after 1, 7, 14, and 21 days. A significant difference was observed between the TCP group and other groups in the days 1 and 7 after cell seeding. This might be due to Ti and Si ion release in the NFTi coated groups (S1: 150 ps, S2: 5 ns, and S3: 30 ns) and Si ion release of the uncoated group in days 1 and 7. Additionally, a similar pH trend was observed on days 14 and 21. However, based on MTS assay and cell culture standards, none of the above pH conditions are toxic for the cells.

### Water contact angle

Contact angles of water droplets on specimens were measured and compared as shown in Fig. 9(e) by increasing laser pulse duration. The CA between the NFTi coating and the water also increased. The uncoated sample with a CA of 53° has the least wettability and the S1 sample has the most wettability with a 25° CA. Significant difference was observed between all of the samples except S3 and TCP.

### Cytotoxicity and cell adhesion

**Figure 10:**
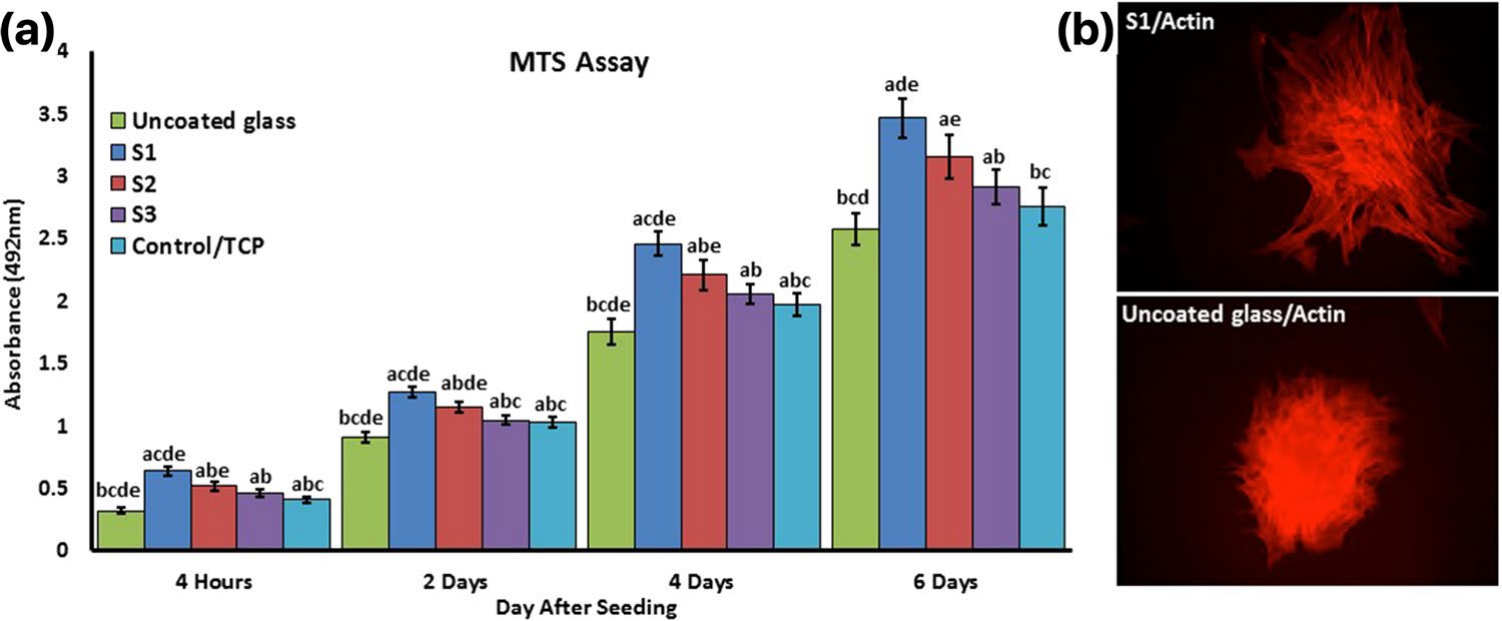
(**a**) Cell attachment 4 hours after seeding and viability MTS assay was used on days 2, 4, and 6. There were significant differences in absorbance over time. a: statistically significant difference compared with uncoated group at *p* < 0.05; (**b**) statistically significant difference compared with S1 group at *p* < 0.05; (**c**) statistically significant difference compared with S2 group at *P* < 0.05. (**d**) Statistically significant difference compared with S3 group at *p* < 0.05; (**e**) statistically significant difference compared with TCP group at *p* < 0.05. (**b**) The cytoskeleton adhesion of BMSCs were stained by phalloidin to observe actin filament expansion in uncoated and S1 samples.

The cytotoxicity and cell adhesion properties of NFTi coatings were determined by the MTS assay of the BMSCs (Fig. 10). Four hours after cell seeding MTS assay was used to show the amount of cell attachment on the different samples. The S1 sample has the highest OD compared to the other samples. The uncoated sample shows the lowest absorbance; the OD of control-TCP was significantly lower than S2, and no significant difference was observed between control-TCP and S3.

To assess cell viability, MTS assay was used on day 2, 4, and 6. The color absorbance quantity for all samples increased continuously with the culture time relative to the number of primary cells adhering, demonstrating a normal growth trend and worthy cytocompatibility. It is clear that all treated samples are better than the untreated glass and TCP. S1 has the highest cell adhesion, viability, and metabolic activity than the others (P < 0.05). The primary adhesion 4 hours after cell seeding is shown in Fig. 10(b), where the BMSCs cultured on the glass sample show a round shape and almost no spreading presents on the surface. In contrast, the complete spreading of BMSCs cultured on S1 is observed.

### Cell migration, F-actin/DAPI staining

**Figure 11:**
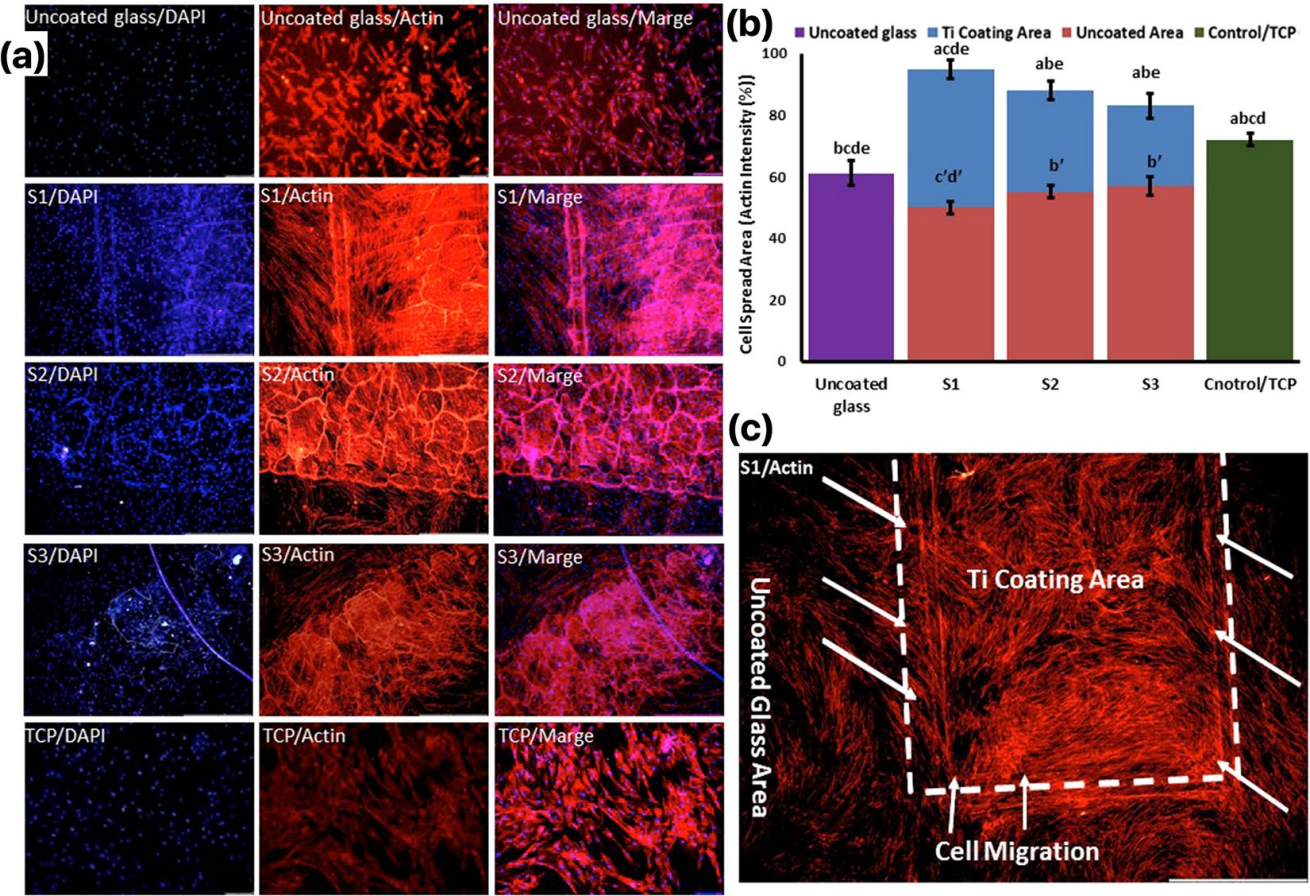
Cells were stained by phalloidin to observe (**a,c**) and quantify (**b**) actin filament expansion and migration in all samples after 7 days. The first column in A is DAPI nuclear staining (blue), second column, phalloidin labelled F-actin (red), and third column, overlaid fluorescent image of immunostained cellular components (merge: DAPI/F-actin). The red bars in B indicate the intensity percentage of actin filament on the glass in S1, S2, and S3 samples and the blue bars describe the intensity percentage of actin filament the NFTi coated area. (**a**) Statistically significant difference compared with uncoated group at *p* < 0.05; (**b**) statistically significant difference compared with S1 group on Ti coating area at *p* < 0.05; (**c**) statistically significant difference compared with S2 group on Ti coating area at *P* < 0.05. (**d**) statistically significant difference compared with S3 group on Ti coating area at *p* < 0.05; (**e**) statistically significant difference compared with TCP group at *p* < 0.05. (**bʹ**) Statistically significant difference compared with S1 group on glass uncoated area at *p* < 0.05; (**cʹ**) statistically significant difference compared with S2 group on glass uncoated area at *P* < 0.05. (**dʹ**) statistically significant difference compared with S3 group on glass uncoated area at *p* < 0.05; (**c**) Higher magnification of BMSCs migration from glass area toward Ti coating area in S1.

Nucleus and actin filament staining was used to show the cell spreading area, cell migration and their morphology on the control samples (glass and TCP) and the NFTi coated samples in the center of an uncoated glass, which can provide a good map of their structures and their orientations in response to the substrates. Figure 11(a) shows the normal morphology of the cells in both the uncoated glass and TCP samples and uniform cell population density along the surface; however, the quantified percentage of cell actin filament was significantly higher in the TCP sample (Fig. 11(b)). The same normal morphology of BMSCs was observed in the NFTi coated samples, but the cell population was centered on the NFTi coats from the uncoated glass. This cell migration shows the S1 sample has the highest significant rate of migration (Fig. 11(c)). Moreover, the intensity of actin filaments in both NFTi coated (blue bars) and uncoated areas (red bars) of the S1, S2, and S3 samples indicates that the highest cell migration rated occurred in the S1 sample, as the actin filament intensity of the uncoated area in the S1 sample is 50 ± 4 (significantly the lowest) and the intensity of this sample in the NFTi coated area is 95 ± 6 (significantly the highest). In other words, all the NFTi coated samples have better staining than the control sample and bare glass, which is a benefit of using NFTi coatings.

### Ions concentration (ICP)

**Figure 12:**
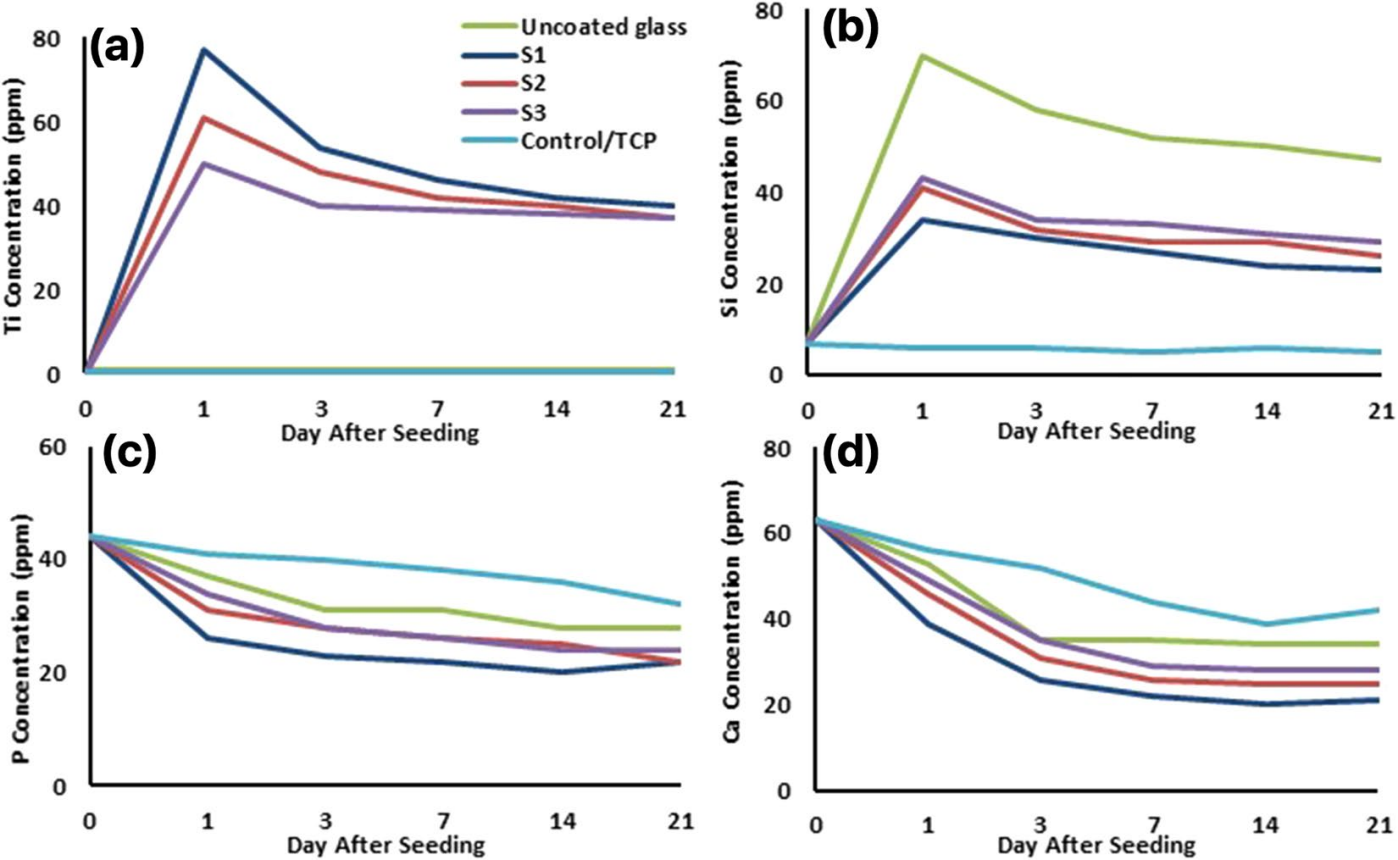
Determining the concentration of released Ti, Si, Ca, and P ions for all samples in culture medium measured by ICP-MS before medium refreshment on days 1, 3, 7, 14, and 21 from cell culture.

Ti, Si, Ca, and P ions concentration of the culture medium were measured before medium refreshment on days 1, 3, 7, 14, and 21 (Fig. 12). The initial concentrations of Si, P, and Ca were 7, 44, and 63 respectively and ion concentration was monitored during the treatment’s duration. Ti^2+^ release was maximum on the first day in all three NFTi coated samples and the highest to lowest Ti^2+^ release was in the S1, S2, and S3 samples respectively. However, the Ti ion concentration of these three samples was almost equal on the last days. More exterior and more active Ti^2+^ was released on the primary days, but this amount decreased due to medium refreshment and less release on the last days.

The amount of 70 ppm Si^4+^ was released by the uncoated glass on the first day, which means that an appropriate amount of Si ion was supplied from the glass in all of the samples. The release amount decreased on the following days due to medium refreshment. The Ti and Si ion release data are exactly reverse in NFTi samples due to greater formation of the Ti layer and less exposure of the Si surface to the solution when the laser pulse duration decreases from 30 ns to 150 ps.

The concentration of both P and Ca ions decreased during the treatment process in all of the samples. The higher to lower decreasing rate of P and Ca ions was observed in the S1, S2, S3, uncoated and TCP samples respectively.

### Osteogenic-related mRNA relative expression

**Figure 13:**
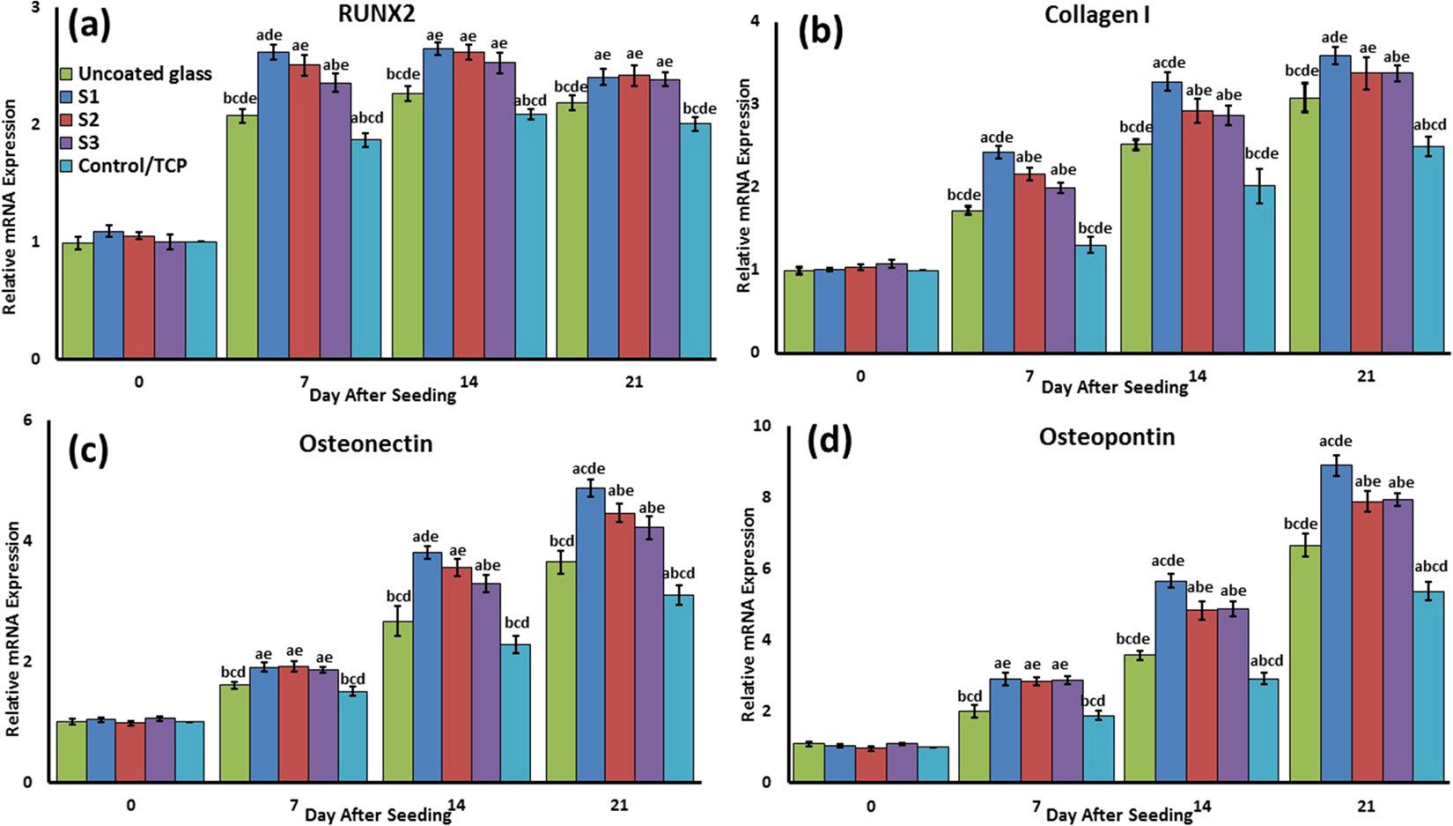
The mRNA relative expression levels of osteogenic genes, included Runx2 (**a**), Collagen I (**b**), Osteonectin (**c**), and Osteopontin (**d**) were determined by qRT-PCR for all samples. a: statistically significant difference compared with uncoated group at *p* < 0.05; (**b**) statistically significant difference compared with S1 group at *p* < 0.05; (**c**) statistically significant difference compared with S2 group at *P* < 0.05. (**d**) Statistically significant difference compared with S3 group at *p* < 0.05; (**e**) statistically significant difference compared with TCP group at *p* < 0.05.

In order to confirm the differentiation of BMSCs, the osteogenic-related mRNA relative expression, including RUNX2, Collagen I, Osteopontin, and osteonectin, was measured by qRT-PCR for different samples. The S1 sample had almost the highest significant relative expression among the different samples along differentiation process time duration for all osteogenic-related genes (Fig. 13). The expression of these to the marker was as follows: S1 > S2 and S3 > uncoated > TCP in all various days of differentiation follow-up. Moreover, the uncoated sample RUNX2, Osteopontin, and osteonectin genes were significantly higher than TCP, especially during the primary stages.

### Mineralization

**Figure 14:**
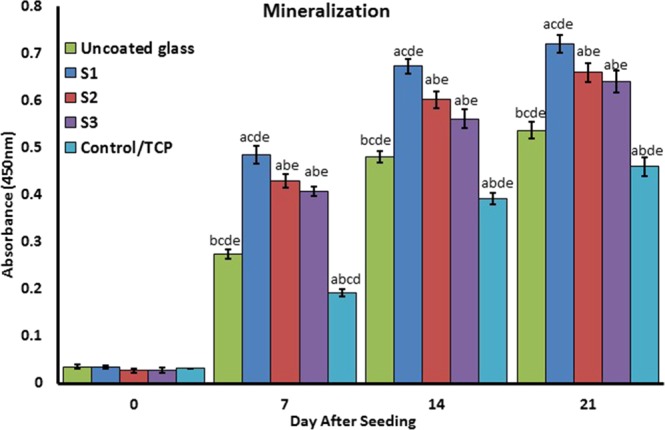
Confirmation of osteogenic differentiation and matrix mineralization of BMSCs by quantification of alizarin red staining. (**a**) Statistically significant difference compared with uncoated group at *p* < 0.05; (**b**) statistically significant difference compared with S1 group at *p* < 0.05; (**c**) statistically significant difference compared with S2 group at *P* < 0.05. (**d**) Statistically significant difference compared with S3 group at *p* < 0.05; (**e**) statistically significant difference compared with TCP group at *p* < 0.05.

To confirm the mineralization of BMSCs, alizarin red staining followed by soluble Ca nodules color absorbance quantification was used, as shown in Fig. 14. The highest to lowest significant color absorbance was as follows: S1 > S2 > S3 > uncoated > TCP. As shown in Fig. 14 and confirmed in the studies, the high level of mineralization occurred on days 7 and 14. Regardless of the differences between laser pulse duration utilized for producing samples, mineralization of NFTi samples generally increased day by day. This shows that all our samples with NFTi coatings have mineralization ability, which can be the result of producing biocompatible NFTi on samples.

### Protein adsorption and biocomplex adsorption/absorption

**Figure 15:**
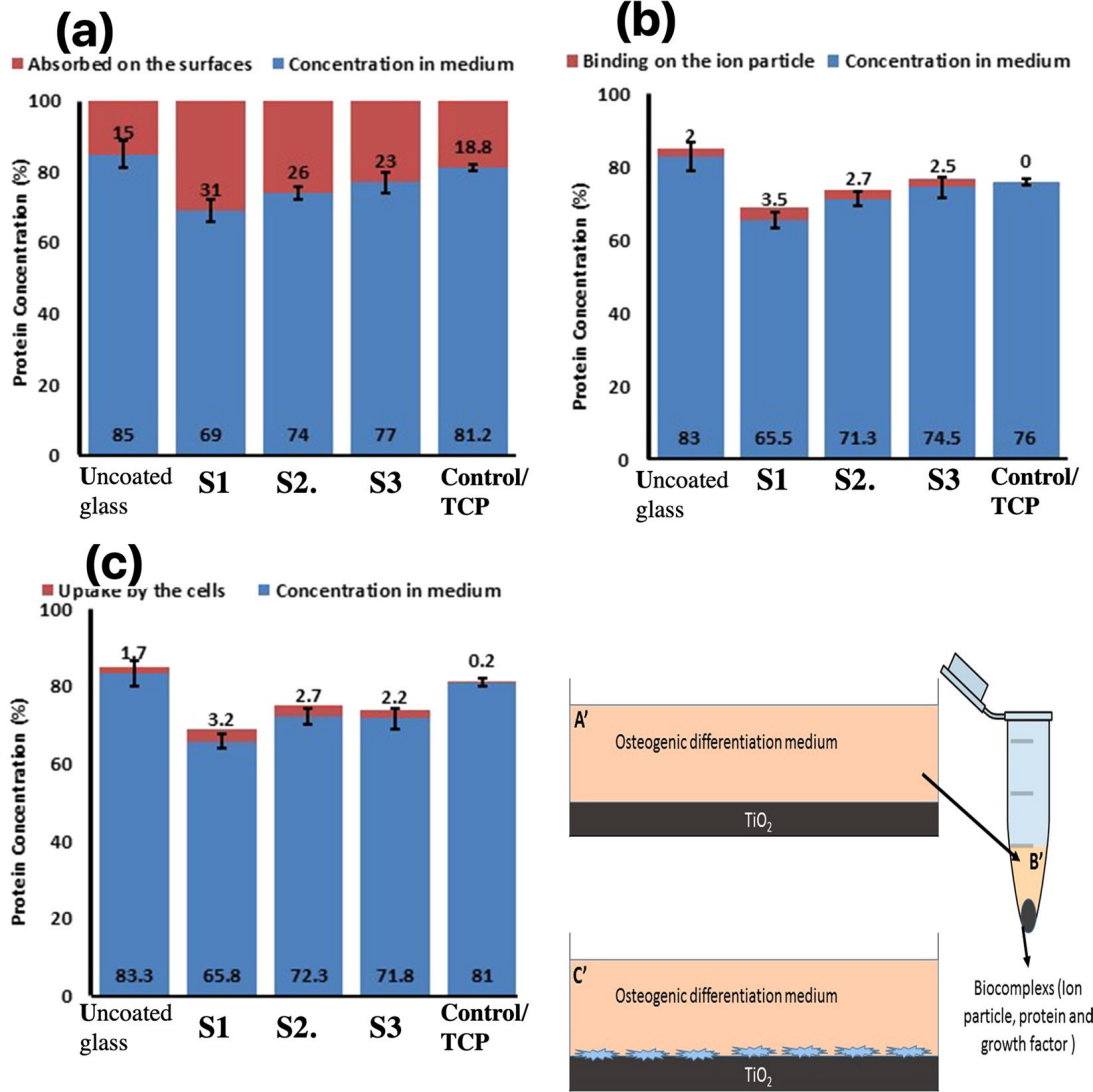
Protein adsorption and biocomplex adsorption/absorption: concentration of protein in osteogenic differentiation medium after 6 hours of immersion (**a**); concentration of protein in osteogenic differentiation medium after 6 hours of immersion and then centrifuged at 14,000 rpm for 30 min (**b**); concentration of protein in osteogenic differentiation medium after 6 hours of immersion with cell culture (**c**).

Surface protein adsorption potential (Fig. 15a), protein-ion biocomplex formation (Fig. 15(b)), and biocomplex cell uptake (Fig. 15(c)) were estimated. Surface protein adsorption (%) for S1, S2, S3, TCP, and uncoated samples were 31, 26, 23, 18.8, and 15 respectively (Fig. 15a).

The highest level of protein-ion biocomplex was observed in the S1 sample. The percentage of biocomplex formation for other samples is as follows: S2 > S3 > uncoated. No biocomplex was formed in the TCP sample because no ion was presented in the sample.

Figure 15c shows the percentage of absorbed biocomplex by the cells. The S1 sample cells had the highest amount of biocomplex absorbance which followed by S2, S3, uncoated and TCP samples respectively.

## Discussion

According to our data, the HLRT is a useful method to achieve high surface bioreactivity, osteogenesis, and osseointegration of NFTi-BMSCs. The S1 sample microtopography (11), surface roughness^19^, wettability^19^, nanofiber-like structures^31^, surface energy^32,33^ and its proteins or biomacromolecules interaction can stimulate cell adhesion^32,34,35^, mineralization and osteogenesis^36^, which leads to faster and more suitable osseointegration *in vitro* and *in vivo*^37^.

The similar pH observed on days 14 and 21 (Fig. 9) could be due to culture refreshments and P and Ca ion release from the saturated hydroxyapatite, which leads to ionic neutralization. It is observed that the pH of the solution including the samples became constant after 14 days of immersion, which can be due to the formation of more NFTi with more SVR as suitable places for deposition of the Ca and P layers. This results in less exposure of NFTi coatings to the culture media by the passive corrosion process^38,39^.

One possible solution for surface modification is to deposit nanostructured artificial biomaterial on the substrate to allow vascularization, protein inherency, proliferation simulation, and accordingly, cell attachment^40,41^. Using nanofiber mesh-like scaffolds is becoming more popular considering that the ECM has the same size nanofiber with interconnected porosity^31^. Titanate nanofibers can provide more nucleation sites for Ca-P precipitation through interconnected microporous surface area production, wettability, and biomolecular absorption. Moreover, a microporous surface promotes nutrition diffusion, vascularization, and blood flow, and this platform can achieve a mechanical intertwining which is particularly beneficial for the biomechanical strength^31,42^.

Liquid droplets deposited on a fiber can have two different behaviors based on the fiber’s surface energy, diameter, droplet surface tension and volume. Larger droplets relative to fibers tend to have a barrel shape and wet the fiber symmetrically. The clamshell conformation for the small droplets relative to fibers can be seen on the fibers and can just wet one side^43^. However, sometimes both types of conformations are seen together. The wettability is in a reductive order due to higher roughness, oxidation, which brings about more surface energy^44–46^: S1, S2, S3 and TCP, and uncoated sample. Rogers has construed that a 12° decrease in the water contact angle of scaffold surface results in a significantly positive response to cell adhesion due to lower surface interaction with toll-like receptors^47^. This indicates that even a slight alternation in the contact angle in our samples could cause a massive neighboring cell response difference in the body.

The trend of the metabolic activity of BMSCs on the samples was around 4 hours, which could be due to the results of primary cell attachment, not the toxicity of the surface. The adhesion and proliferation of BMSCs on NFTi coated glass significant increased by decreasing laser pulse duration, indicating that the NFTi prepared by the shortest laser pulse duration (150 ps) receives more suitable substrate for cell culture. Moreover, the F-actin filament immunofluorescent staining shows the higher cell spreading and cell attachments of the S1 sample compare to the uncoated sample. The higher cell adhesion to the NFTi coated surface is due to topography, roughness, porosity, wettability, surface energy, and protein adsorption/absorption. These data illustrate the higher biocompatibility interaction between cell and the coated surface even in comparison with the standard surface of cell culture (TCP).

According to schematic Fig. 1 OH^−^ groups on the surface increase due to HLRT process. Also, considering previous studies about the corrosion of Ti in body fluid, the corrosion reaction is initiated by conversion of Ti to Ti^2+^ and H_2_O to oxygen and OH^−^. Titanium hydroxide layers are then formed from the free titanium and the hydroxyl ions. Consequently, chloride ions from the culture media diffuses into NFTi and forms titanium chloride; these would then be hydrolyzed into hydroxide and free acid and would consequently decrease the pH of the solution (Fig. 9D)^17,38,39^. Based on Yu *et al*., these OH^−^ groups which were formed on the UV modified NFTi surface can increase the adsorption of α-helix and β-sheet structures of the adhesion related proteins including fibronectin, laminin, vitronectin, and elastin^48^. Such proteins are naturally found in plasma and assist the cells to adhere to the extracellular matrix. The same mechanism may happen due to the medium proteins.

The stained F-actin images indicate that many F-actin filaments on differently treated samples migrated well toward the 3D titanate coat area. Sample S1 has the highest migrations. It can also be concluded that the stained cytoskeleton migration to the substrate decreases by increasing the pulse duration and shows the minimum migration in contact with bare glass and control samples. All these results can be due to the cells’ ability to spread over NFTi coatings, a known response sign^4,49^ that can be distinguished via immunofluorescent staining such as DAPI/Actin staining. Consequently, the NFTi coating produced by the shortest laser pulse duration is the best surface for cells to be spread in comparison with others. This NFTi coating produced with 150 ps pulse duration has more fibers and more SVR, which are beneficial for cell propagation^13^. Natural MSCs cell migration to the damaged tissues is the basis of specific cell differentiation and tissue healing^50^. The fundamental property of surface implant preference in bone tissue engineering is appropriate cell migration toward that surface, which is called osseointegration^51^. The S1 sample had the best osseointegration. Cell migration based on its adhesion markers on the surface has recently been explained by haptotaxis mechanism^52^. Haptotaxis, a subclass of chemotaxis, is the directional motility of cells and is a key factor in the regeneration of injured tissues^53^. MSCs can navigate through the tissue using tactile cues based on the gradients supplied by ECM or artificial biomaterial surface such as biochemical and biomechanical properties of the surface such as topography, roughness, wettability, and surface energy^52^. Actin cytoskeletal regulatory proteins are considered important components of haptotaxis in cell migration and cell spreading^54–56^. However, the detail mechanisms of this process are yet to be studied. As an example, the biocomplex adhesion to the extracellular receptors, e.g., integrin, can possibly initiate the proliferation, migration and differentiation signaling pathways through a protein e.g., irisin^57^, which was carried by the biocomplex^34,51,53,58^.

Higher amounts of Ti^2+^ and the highest ionization rate occurred in the S1 samples due to higher laser impulse, so a higher amount of ion release was observed in the S1 sample. Moreover, based on the Fig. 12 more active Ti^2+^ leads to more biocomplex formation and more Ti^2+^ absorption by the surface and the cells which increase cell differentiation. The charts in Fig. 12a,b show that in all three coated samples the amount of Si release was about 50% less than in the uncoated glass. This means that NFTi coat can possibly create corrosion resistance for the lower layer of the coat, suggesting that HLRT-produced coat can be used for titanium alloys and stainless steel-based implants. Although the mechanical properties of titanium alloys and the stainless steel-based implants are acceptable, they are not generally used due to the release of noxious ions including Cr, Ni, and others. Not only did the amount of NFTi increase by decreasing the laser pulse duration, but the SVR of NFTi increased, which leads to the nucleation and growth of more Ca and P elements from culture media, as shown in Fig. 12c,d ^17,18^. Generally, in synthesis of bone-graft substitutes, formation of HA-like layers on substrates brings them into better connection with surrounding living tissue^20,59,60^. Thus, surface modification for better calcium phosphate formation is vital to minimize toxicity and lower the possibility of rejection due to moving or loading glass-coated implants while *in vivo*^61,62^. The deposition of Ca and P ions (Fig. 12) and alizarin red (Fig. 14) specifies that mineralization, and as a result osteogenic differentiation, occurred sooner in the S1 sample. Near the middle of the treatment process, the soluble Ca and P ions and the hydroxyapatite sediment reach a balance. This is in line with previous studies which mentioned that the highest rate of mineralization occurs in the initial days^36^.

In the gene expression results, the following genes were chosen from various stages of osteoblast differentiation. RUNX2, an osteogenic transcription factor, high expression on day 7 shows the initial steps of the differentiation process, as the highest level of RUNX2 expression is normally observed during G1 stage^63^. This indicates that the pre-osteoblast differentiation occurred in a higher grade in the S1 sample than the others. In addition, collagen I, the osteoblast specific marker, is expressed during the differentiation process, which indicates that cell matrix secretion is higher in the S1 sample. Osteopontin and osteonectin are two of the markers expressed in mature osteoblasts. Also, gene expression in the uncoated sample was significantly higher than TCP especially during the primary stages. This could be due to an increase in the protein adsorption/absorption by the surface and biocomplex formation in the uncoated sample, so it is probable that it is acting as a bioglass for osteogenic differentiation. As illustrated in Fig. 14, the NFTi structures on the substrate can attract agents such as calcium from the surrounding environment; this results in higher bioactivity, good stability with the human body (if implanted), and less chance of rejection when in contact with living tissue^18,30,38^. It is clear that decreasing the laser pulse duration brought about more mineralization. Surface energy, porosity, roughness, wettability, and protein adsorption/absorption lead to an increase in the Ca nodules, and as a result osseointegration is developed^19^.

In addition, proteins, growth factors, and cytokines adsorption and absorption activate the signaling pathways of the cells, which leads to various alternations in the cellular mechanisms including division and differentiation. The receptors of these proteins appear mainly on the surface of the cells; however, some receptors might be entrapped in the cell cytoplasm, and to activate the proteins need to enter the cell^64^. The biocomplexes which entrapped proteins, growth factors, and cytokine uptake probably initiate osteogenic differentiation mechanisms in an osteogenic differentiation medium^65^. The same mechanism may occur in the bone tissue environment^66^. Our data illuminates that NFTi coats increase the rate of osteogenic differentiation and promote cell adhesion. Interestingly, our findings indicate that the uncoated sample induces more osteogenic differentiation compared with the TCP; however, the cell adhesion was significantly higher in the TCP sample compared with the uncoated glass. This finding confirms that the Ti^2+^ ^51,67,68^ and Si^4+^ ^69^ release significantly promote the differentiation through proteins, growth factors, and cytokines biocomplex formation. In has been confirmed that Si ions play a vital role in the mineralization and osteogenesis process (79). Studies have shown the beneficial biological effects of Ti^2+^ ions on the viabilities of osteoblast and osteoclast and bone remodeling^67,70^. Degabriel has thoroughly studied the mechanisms of various protein surface adsorption and ion absorption including the kinetics, chemophysic, and thermodynamic of the mechanism^71^.

Lee *et al*. found that on hydrophilic surfaces protein adsorption has better binding with transmembrane receptors than hydrophobic surfaces, which facilitates cell-ECM adhesion and in fact supports more binding to cells. They recognized that the orientation and conformation of adsorbed protein on the surface which contains OH^−^ may consequently form stronger bonds with integrin receptors^72^. Also, the biocomplex will enter the cell by endocytosis (absorption) and will reach the appropriate intracellular receptor by the guidance of motor proteins through the cell trafficking process^73^. When an exterior particle is endocytosed, several mechanisms could comprise the processing of that material such as targeting lysosome, phagosome, and autophagosome, which is associated with the regulation of cell signaling including autophagy as a probable cellular intermediate^53,68,74–76^. Recent studies have found that titanate, gold, silica, or iron oxide NPs may initiate the autophagy process^69^. The results of the Thiele studies indicate that protein adsorption on the particle’s surface can significantly increase their phagocytosis^77^. Ha *et al*. suggested that Si NPs stimulate osteoblast differentiation and mineralization by the auto phagosome formation via ERK1/2 MAP pathway *in vitro* and preferable bone remodeling *in vivo*^69^. Hou *et al*. revealed that TiO_2_ NPs spreading from a titanium implant surface can affect the biological function of the neighboring cells in two ways: neighboring cell protein adsorption alternation and direct cell absorption^67^. The exposure of nanoparticle to body fluids leads to nanoparticle embracement by 50 different types of proteins, which is called biocomplex. TiO_2_ biocomplexes were found to be ideal for osteogenic differentiation induction of ADSCs, which was mediated by osteogenic differentiation-specific markers, i.e., OCN and RUNX2 by epigenetic modifications^78^. In addition, it was declared that TiO_2_ biocomplexes could be effective in the cell attachment and spreading which promoted the osteogenic differentiation by focal adhesion kinase phosphorylation^79^. Another bioinformatics-based approach studied the role of some enzymes and ion pumps in TiO_2_ NP-induced BMSCs to osteoblast differentiation^80^. It has also been verified that TiO_2_ biocomplexes have been used as a nanocarrier for the loading and control of osteogenesis growth factors i.e., BMP2 release^75^. It is therefore necessary to evaluate the mechanism of Ti NPs in osteogenic differentiation. Among all of the treated surface, the S1 sample can obviously generate the best surface bioreactivity, which results in early osteogenic differentiation of BMSCs. These HLRT-made NFTi surface implants meliorate the osseointegration.

## Conclusion

Synthetic bone implants along with stem cells are a new approach in bone regeneration therapy or replacement. The advantages of titania make it a desirable option for orthopedic implant materials. Modern surface modification strategies improve the bioreactivity of these surfaces and their osteogenesis, which results in a more suitable osseointegration. In this investigation, we presented a new approach that has not been used in biomedical applications. This cost-effective method can provide a metallic nanofiber structure surface that can be coated on multiple surfaces to be used in a variety of applications. We strongly believe the proposed HILIRT method can open new doors to opportunities for fabrication of better surface bioreactivity materials especially in terms of cell adhesion, proliferation, and osteogenic differentiation, promising to improve the wider range of bone tissue regeneration applications including implant fabrication, LOC, and bio-MEMS devices. Our findings define the beneficial effects of this method both in experimental research conditions to improve the quality of osteogenesis and in the medical osseointegration application as an implant.
